# The flavin monooxygenase Bs3 triggers cell death in plants, impairs growth in yeast and produces H_2_O_2_ *in vitro*

**DOI:** 10.1371/journal.pone.0256217

**Published:** 2021-08-19

**Authors:** Christina Krönauer, Thomas Lahaye

**Affiliations:** 1 Department of Molecular Biology and Genetics, Aarhus University, Aarhus C, Denmark; 2 University of Tübingen, ZMBP–General Genetics, Tuebingen, Germany; Universidade de Lisboa Instituto Superior de Agronomia, PORTUGAL

## Abstract

The pepper resistance gene *Bs3* triggers a hypersensitive response (HR) upon transcriptional activation by the corresponding effector protein AvrBs3 from the bacterial pathogen *Xanthomonas*. Expression of *Bs3* in yeast inhibited proliferation, demonstrating that Bs3 function is not restricted to the plant kingdom. The Bs3 sequence shows striking similarity to flavin monooxygenases (FMOs), an FAD- and NADPH-containing enzyme class that is known for the oxygenation of a wide range of substrates and their potential to produce H_2_O_2_. Since H_2_O_2_ is a hallmark metabolite in plant immunity, we analyzed the role of H_2_O_2_ during Bs3 HR. We purified recombinant Bs3 protein from *E*. *coli* and confirmed the FMO function of Bs3 with FAD binding and NADPH oxidase activity *in vitro*. Translational fusion of Bs3 to the redox reporter roGFP2 indicated that the Bs3-dependent HR induces an increase of the intracellular oxidation state *in planta*. To test if the NADPH oxidation and putative H_2_O_2_ production of Bs3 is sufficient to induce HR, we adapted previous studies which have uncovered mutations in the NADPH binding site of FMOs that result in higher NADPH oxidase activity. *In vitro* studies demonstrated that recombinant Bs3_S211A_ protein has twofold higher NADPH oxidase activity than wildtype Bs3. Translational fusions to roGFP2 showed that Bs3_S211A_ also increased the intracellular oxidation state *in planta*. Interestingly, while the mutant derivative Bs3_S211A_ had an increase in NADPH oxidase capacity, it did not trigger HR *in planta*, ultimately revealing that H_2_O_2_ produced by Bs3 on its own is not sufficient to trigger HR.

## Introduction

Programmed cell death (PCD) provides protection against biotrophic microbial pathogens and is a hallmark of plant immune reactions. Execution of pathogen-triggered plant cell death, often referred to as the hypersensitive response (HR), is generally controlled by two distinct immune receptor classes, membrane-resident pattern recognition receptors (PRRs) and intracellular nucleotide-binding domain leucine-rich repeat (NLR) proteins, which are the most abundant type of plant resistance (*R*) proteins [1]. Upon recognition of pathogen structures or pathogen-induced changes in the host cell, these receptors initiate a number of cellular events, like calcium influx, burst of reactive oxygen species (ROS), and accumulation of salicylic acid (SA) that are assumed to serve as signal molecules that eventually trigger HR [2]. It is unclear, however, how the activation of plant immune receptors eventually translates into a cell death reaction.

We study the *R* gene *Bs3* from pepper (*Capsicum annuum*) which mediates recognition of the *Xanthomonas* transcription activator like effector (TALE) protein, AvrBs3 [2]. TALEs are one class of bacterial effectors that, upon injection into host cells, translocate to the plant nucleus where they bind to an approximately 20 base pair long effector binding element (EBE) and transcriptionally activate the downstream host susceptibility (*S*) gene to promote disease [3]. Some genotypes of otherwise susceptible plant species contain TALE-compatible EBEs upstream of transcriptionally controlled cell death executor genes. TALE-induced transcriptional activation of these EBE-containing executor alleles triggers HR and thereby stops proliferation of the biotrophic pathogen *Xanthomonas*. Accordingly, executor alleles with TALE-compatible EBEs act as plant *R* genes although the intrinsic function of executor genes might lie in other processes, such as for example developmentally-regulated cell death.

Executor-type *R* genes have distinct functional modules for effector recognition (*R*-gene promoter) and orchestration of the immune response (executor R protein). This is in striking contrast to constitutively expressed NLR type R proteins, which are known to mediate both, detection of effectors and induction of an immune reaction. At the mechanistic level, there are resemblances between executor type R proteins and activated NLRs since both trigger HR. However, whether or not executors employ canonical immune pathways that are utilized by NLRs needs to be elucidated.

Six executor-type *R* genes have been cloned so far [2, 4–8]. With exception of the rice executor R proteins Xa10 and Xa23 that share about 50% sequence identity, executor R proteins show neither sequence relatedness to each other nor homology to any other NLR-type or PRR-type immune receptor. Within the class of executor R proteins, Bs3 is exceptional as it is the only one that shares homology to a protein class of known function. More specifically, Bs3 shows homology to flavin-containing monooxygenases (FMOs), a class of enzymes that uses molecular oxygen (O_2_) for oxygenation of metabolites [9]. In plants, FMOs are a large and diverse group, but only a few members that have been found to function in hormone production or pathogen defense have been characterized so far [10–12]. Bs3 is most closely related to YUCCA proteins, a plant-specific FMO subgroup that catalyzes the final step in tryptophan-dependent auxin (Indole-3-Acetic Acid; IAA) biosynthesis [10] but despite its similarity to YUCCA, *Bs3* expression does not lead to increased auxin levels [13]. The most prominent difference of Bs3 and YUCCAs is a stretch of ~70 amino acids, that is conserved across all YUCCAs but absent from Bs3 and which harbors a endoplasmic reticulum anchor sequence that can not be exchanged in between YUCCAs and Bs3 without loss of protein function [13]. *Bs3* expression coincides with the accumulation of the immunity related metabolites salicylic acid and pipecolic acid [13]. Yet, in contrast to other plant FMOs that have a function in immune-signaling or chemical defense [14], Bs3 is unique as it is the only FMO known to trigger cell death. Homology of Bs3 and the well-studied FMOs provides a unique opportunity to establish testable biochemical models of how Bs3 triggers HR.

Given that Bs3 structurally resembles FMOs, we tested if Bs3-induced cell death can be explained by the well-studied FMO enzymatic cycle. Evidently, reduction of the bound FAD cofactor in FMOs by NADPH and binding of molecular oxygen results in a C4a-(hydro) peroxyflavin (C4a) intermediate, which is ready to oxygenate suitable substrates. If no metabolic substrate is available, the FMO C4a intermediate breaks down without substrate oxygenation, a process referred to as the uncoupled reaction, where reduction equivalents are released as H_2_O_2_ [15]. While it is unclear if H_2_O_2_ production by FMOs via the uncoupled reaction serves a biological function, the role of H_2_O_2_ as a signaling molecule in plant immune reactions is well established [16, 17]. We therefore speculated that unlike YUCCAs, which bind and oxygenate IPA, Bs3 does not oxygenate a metabolic substrate but instead acts exclusively as an NADPH oxidase that produces H_2_O_2_ to trigger HR.

In this study, we show that *Bs3* expression not only triggers HR in plant cells but also inhibits proliferation of yeast cells. *In vitro* studies of recombinant Bs3 protein and *in planta* studies with the redox-sensitive roGFP2 suggest that Bs3 oxidizes NADPH and produces H_2_O_2_. To analyze if H_2_O_2_ released via a Bs3 uncoupling reaction is causal for HR, we created a Bs3 derivative that due to a mutation in its NADPH binding site has elevated NADPH oxidase activity. While, this Bs3 derivative showed elevated NADPH oxidase activity *in vitro*, its expression did not affect yeast growth and failed to trigger HR *in planta*. These observations are not consistent with a model where ROS produced by Bs3 is the metabolic trigger of Bs3-dependent HR and suggest that Bs3 converts a so far unknown substrate to trigger cell death.

## Results

### Bs3 but not Bs3_S211A_ triggers HR *in planta* and growth arrest in yeast

**Fig 1:**
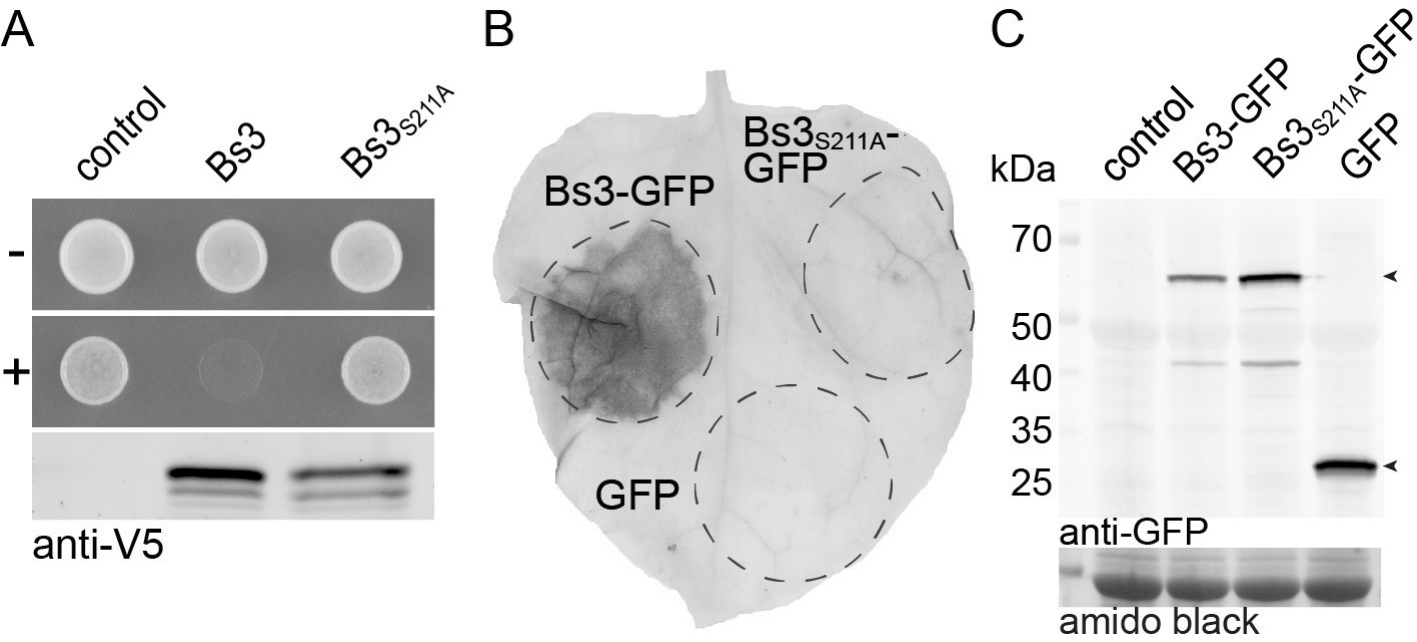
Bs3 but not Bs3_S211A_ induces HR in plants and growth arrest in yeast. A) *S*. *cerevisiae* carrying Bs3-V5 or Bs3_S211A_-V5 under control of the inducible *Gal1* promoter was dropped onto repressing (-) or inducing (+) medium and incubated at 28°C for two days. Protein expression was monitored in liquid culture eight hours after induction via an anti-V5 Western Blot B) *35S* promoter driven T-DNA constructs encoding Bs3-GFP, Bs3_S211A_-GFP or GFP were delivered into *N*. *benthamiana* leaves via *Agrobacterium*-mediated transient transformation. At 4 dpi, the leaf was harvested and cleared in ethanol. The HR is visible as dark spot. C) *In planta* expression of T-DNA-encoded proteins was monitored via an anti GFP immunoblot. Amido black staining was used to visualize total protein load.

Previous studies on the rice executor-type R protein Xa10 from rice uncovered that Xa10 triggers cell death not only in plant cells but also in human cells [6]. Inspired by this finding we wondered whether function of the executor R protein Bs3 is restricted to plants or if it would be functional in the budding yeast *Saccharomyces cerevisiae*, a model system of eukaryotic genetics. To study Bs3 function in yeast, we cloned the wild-type *Bs3* gene into a yeast expression vector under control of a galactose inducible promoter (*pGAL1*). Yeast transformants were grown in liquid medium, dropped onto agar plates containing either glucose (repressing) or galactose (inducing) and incubated at 28°C for two days. We found that yeast containing wild-type *Bs3* grew only on glucose- but not on galactose-containing agar plates, suggesting that *Bs3* expression inhibits proliferation of yeast cells (Fig 1).

FMO function generally depends on transient binding of the NADPH cofactor, and mutations in codons that translate into conserved glycine residues within the NADPH binding site (S1 Fig) of FMOs have been shown to cause loss of enzyme activity [18, 19]. Mutational studies also uncovered specific mutation types within the NADPH binding site of FMOs that lead to derivatives producing increased amounts of H_2_O_2_ relative to the corresponding wild-type protein. For example, a serine to leucine change within the NADPH binding site of human FMO2 (GxGx**S**G → GxGx**L**G) or a serine to alanine change within the NADPH binding site of the *Aspergillus fumigatus* FMO SidA (GxGx**S**G → GxGx**A**G), result in a derivative with increased NADPH oxidase activity [16, 20]. The respective serine residue within the NADPH binding site is present in Bs3 and all Arabidopsis and pepper YUCCA proteins (S1 Fig). We aimed to echo such mutations in the context of the pepper Bs3 protein to generate a mutant derivative with increased NADPH oxidase activity that would possibly induce a faster Bs3 HR. To do so, we mutated the triplet encoding the serine at residue 211 to an alanine codon, to create Bs3_S211A_, a Bs3 derivative which would conceivably produce increased H_2_O_2_ levels. To test whether or not Bs3_S211A_ triggers HR *in planta*, we agroinfiltrated *35S* promoter driven *Bs3-GFP* or *Bs3*_*S211A*_*-GFP* into *N*. *benthamiana* leaves. These agroinfiltration assays showed that Bs3, but not Bs3_S211A_ triggered HR in *N*. *benthamiana* leaves (Fig 1). Immunoblot analysis showed that the Bs3-GFP and Bs3_S211A_-GFP fusion proteins were equally abundant *in planta* suggesting that the S211A mutation affects Bs3 function but not protein stability (Fig 1). Correspondingly, we expressed *pGal* driven *Bs3*_*S211A*_ *in S*. *cerevisiae*. No growth defect of the strain carrying *Bs3*_*S211A*_ could be observed on inducing medium after two days of incubation at 28°C (Fig 1). Therefore, the serine to alanine mutation within the NADPH binding site resulted in a non-functional Bs3 derivative that does neither trigger HR *in planta* nor inhibit growth of yeast cells.

### Execution of Bs3 HR correlates with accumulation of H_2_O_2_ *in planta*

**Fig 2:**
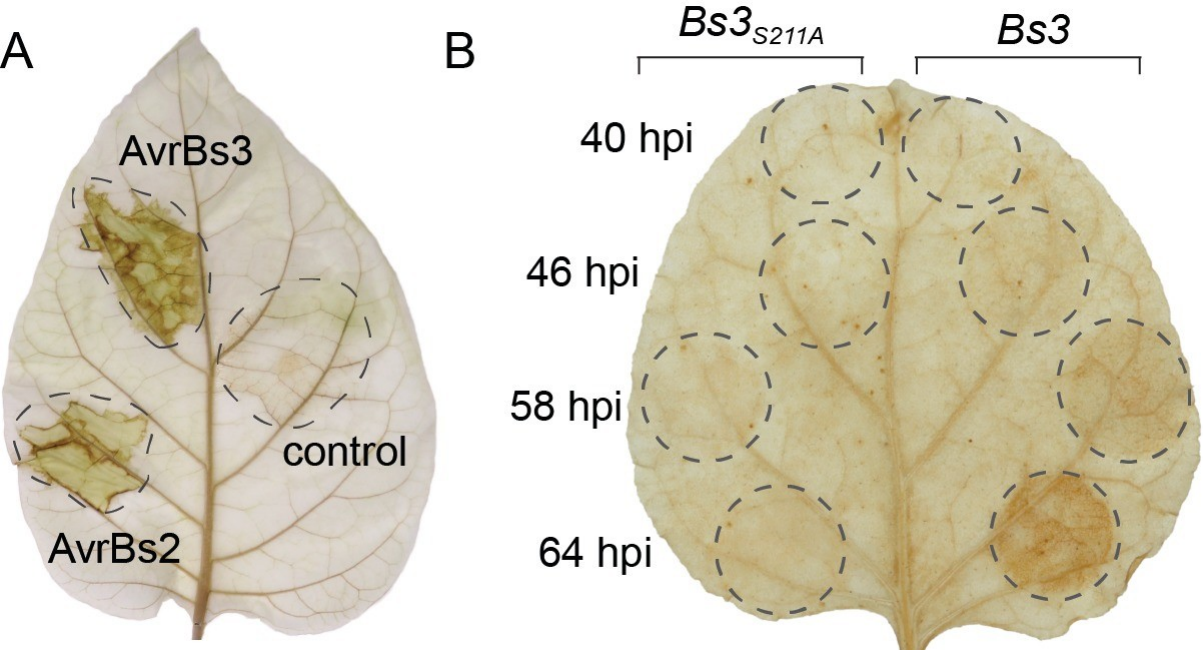
The Bs3-dependent HR correlates with H_2_O_2_ accumulation. A) Leaves of the *Capsicum annuum* genotype ECW123, which contains the bacterial spot (*Bs*) *R* genes *Bs2* and *Bs3* were infiltrated with the *Xanthomonas euvesicatoria* strain 82–8 uns or corresponding transformants delivering the effector proteins AvrBs2 or AvrBs3. 30 hpi the inoculated leaf was stained with 3,3-diaminobenzidine tetra-hydrochloride (DAB) solution and cleared with ethanol. Dashed lines indicate inoculated leaf sections. B) *35S* promoter driven *Bs3-GFP* and *Bs3*_*S211A*_*-GFP* T-DNA constructs were infiltrated into *N*. *benthamiana* leaves at 0h, 6h, 18h and 24h. 64 hours after the first infiltration, the leaf was detached and vacuum infiltrated in DAB solution to visualize H_2_O_2_ accumulation. Leaf was de-stained in hot ethanol. Dashed lines mark infiltrated areas.

To clarify whether or not occurrence of Bs3 HR would correlate with accumulation of H_2_O_2_ within infected tissue, we performed histochemical staining of pepper leaves using 3,3-diaminobenzidine tetrahydrochloride (DAB) as described previously [21]. We infected the pepper variety Early Calwonder 123 (ECW123; [22]) that contains the bacterial spot (*Bs*) plant *R* genes *Bs2* and *Bs3* with a *Xanthomonas euvesicatoria* (*Xeu*) strain that lacks the *avrBs2* and *avrBs3* genes (82-8 universally susceptible [uns]) or isogenic transconjugants containing either *avrBs2* or *avrBs3*. Brown staining, indicative of accumulation of H_2_O_2_, was observed in leaf tissue infected with *Xeu* strains delivering either AvrBs2 or AvrBs3 after 30 hours (Fig 2). The DAB staining shows that the execution of an HR by the NLR protein Bs2 or the executor R protein Bs3 in pepper leaves both correlate with a local increase of H_2_O_2_ levels. Similarly, HR induced by *Agrobacterium tumefaciens* mediated delivery (agroinfiltration) of a *35S* promoter-driven *Bs3* T-DNA in *Nicotiana benthamiana* leaves correlates with DAB staining in *N*. *benthamiana* leaves that becomes visible two days after inoculation (Fig 2 [13]). Our studies in pepper and *N*. *benthamiana* are compatible with a model in which Bs3 triggers HR via production of H_2_O_2_. However, it cannot be determined by histochemical staining whether H_2_O_2_ is directly produced by Bs3, or if a Bs3-dependent immune pathway eventually results in expression or activation of proteins that produce H_2_O_2_.

### Recombinant Bs3 and Bs3_S211A_ proteins bind FAD

**Fig 3:**
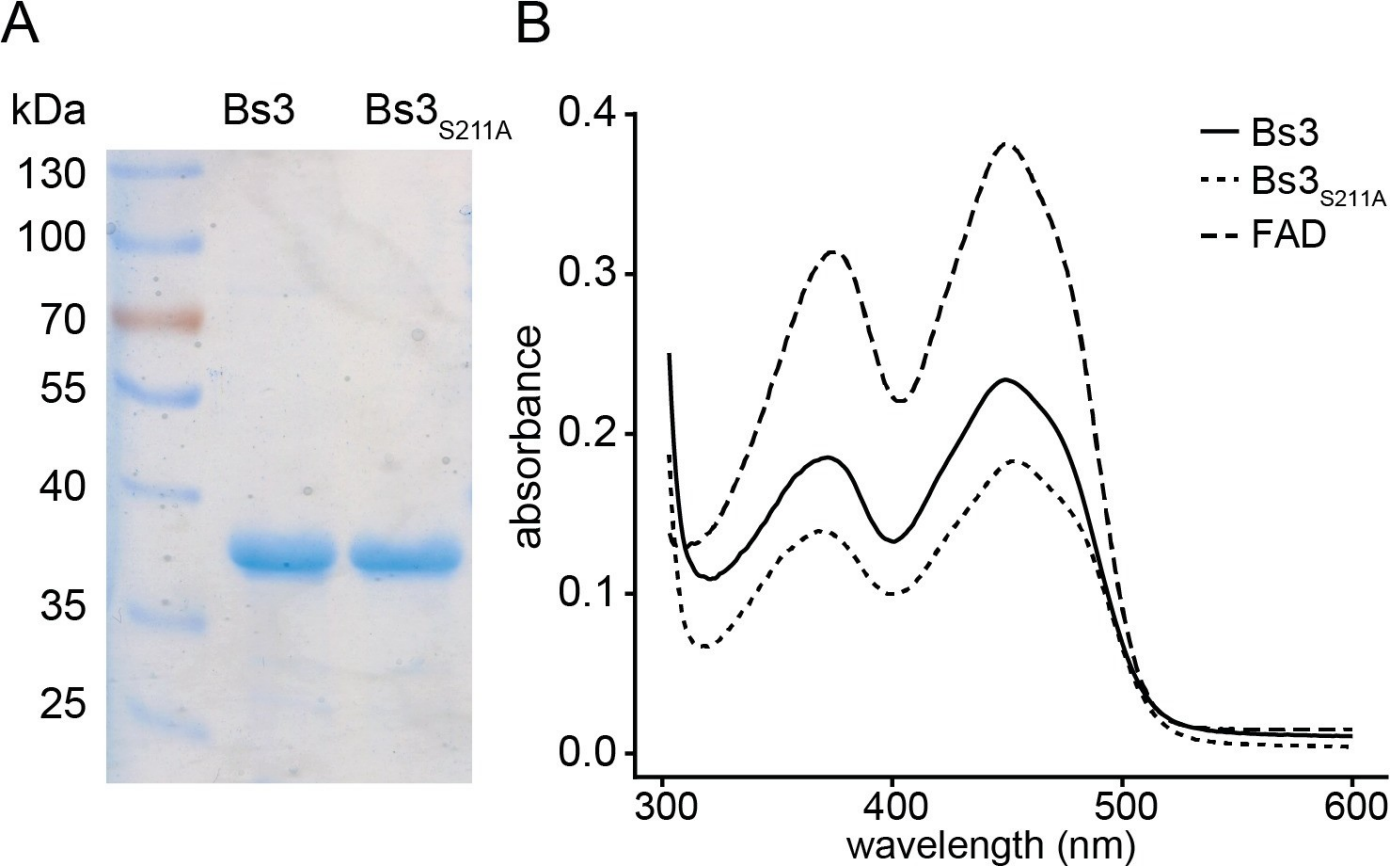
Recombinantly expressed Bs3 and Bs3_S211A_ bind the cofactor FAD. A) Recombinant Bs3 and Bs3_S211A_ proteins were expressed in *E*. *coli*, affinity purified and loaded onto an SDS polyacrylamide gel. B) UV-vis spectrum of FAD (dashed line), Bs3 (solid line) and Bs3_S211A_ (dotted line).

Plant immune reactions generally correlate with the release of H_2_O_2_ [17, 23]. Accordingly, our observation that Bs3-triggered HR correlates with release of H_2_O_2_ (Fig 2) does not clarify if the detected ROS is produced by Bs3 itself, or by a potential downstream immune signaling component that Bs3 recruits to trigger HR. To clarify if Bs3 indeed has NADPH oxidase activity, we studied recombinantly-expressed *Bs3* by *in vitro* studies. *Bs3* and *Bs3*_*S211A*_ were expressed in *E*. *coli* and soluble Bs3 and Bs3_S211A_ protein were affinity purified with yields of two milligram per liter of culture (Fig 3). Given that Bs3 has homology to FMOs, we expected that a bound FAD cofactor was crucial for enzymatic activity [24]. Indeed, supplementation of the lysis buffer with FAD cofactor was necessary to obtain active Bs3 protein and to increase protein yield. In contrast to the purification protocol established for the *Arabidopsis thaliana* YUC6 protein, which uses extraction buffers containing 0.5 M sodium chloride [10], Bs3 purification required low sodium chloride conditions (< 100 mM). The purified Bs3 and Bs3_S211A_ proteins were bright yellow and the UV-vis spectra show characteristic peaks similar to FAD. Notably, we observed a slight shift of the local maxima of Bs3_S211A_ (at 368 nm and 453 nm) compared to the wild type Bs3 protein (373 nm and at 448 nm; Fig 3). This observation is consistent with the expected slight structural changes in Bs3_S211A_ as compared to wild-type Bs3.

### Bs3_S211A_ has higher NADPH oxidation activity and produces more H_2_O_2_ than Bs3 *in vitro*

**Fig 4:**
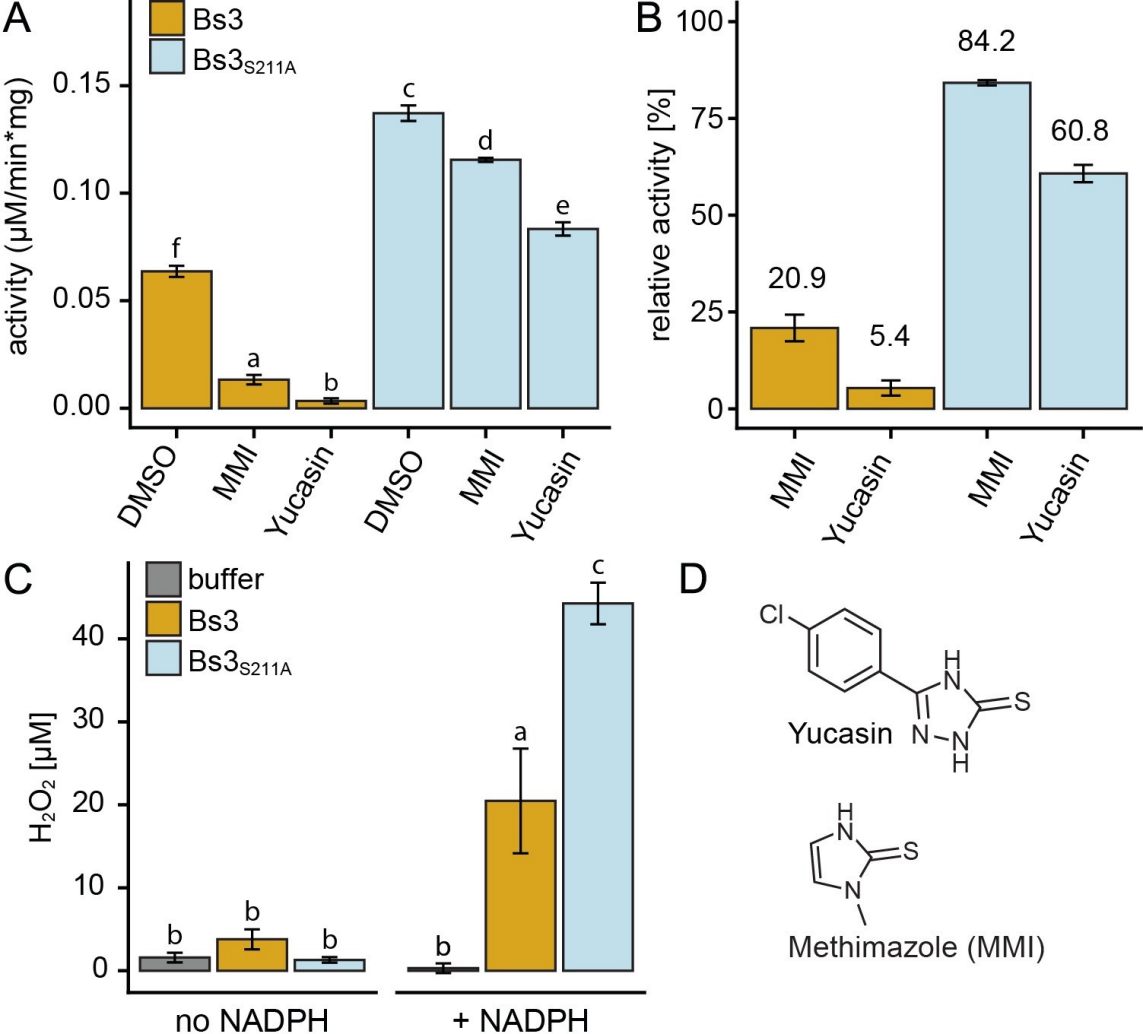
NADPH oxidation and H_2_O_2_ production by Bs3 and Bs3_S211A_. A) Buffer containing 100 μM NADPH was mixed with 0.2 μM Bs3 or Bs3_S211A_ and incubated at 25°C. NADPH oxidation was monitored via decrease of absorbance at 340 nm. Yucasin and methimazole (MMI) were dissolved in DMSO and added at a final concentration of 50 μM. B) Values of Bs3 and Bs3_S211A_ samples containing MMI and yucasin (from A) were normalized to DMSO control to show the relative activity compared to the non-treated (DMSO) sample. C) 0.4 μM of protein was mixed with buffer containing no or 100 μM NADPH and incubated for five minutes at RT. The samples were subsequently mixed with HyPerBlu in a 1:1 ratio to measure H_2_O_2_ concentrations. Luminescence is measured after 10 min of incubation. Bars indicated mean +/s SD of three replicates. D) Chemical structures of yucasin and methimazole (MMI). Different letters denote statistically significant differences (P < 0.05, ANOVA with posthoc Tukey Honest Significant Difference test).

To compare NADPH oxidase activities of recombinant Bs3 protein and its mutant derivative Bs3_S211A_, we mixed corresponding protein fractions with the cofactor NADPH and monitored NADPH consumption via spectrophotometric measurements at 340 nm. The concentration of active protein was calculated from absorbance at 450 nm and the extinction coefficient of FAD (ε = 11300 M^-1^ cm^-1^). At 25°C, we measured an NADPH oxidation activity of 63 nmol/mg*min for Bs3 and 137 nmol/mg*min for Bs3_S211A_ (Table 1 and Fig 4). These *in vitro* studies show that the Bs3_S211A_ mutant has approximately two-fold higher NADPH oxidase activity compared to that of the Bs3 wild type protein. This is in accordance with our expectation that the mutation of the conserved serine to alanine within the NADPH binding site destabilizes the C4a intermediate of Bs3_S211A_ and favors release of H_2_O_2_.

**Table 1:** Yucasin and methimazole (MMI) decrease NADPH oxidase activity of Bs3 and Bs3_S211A_.

Sample	Compound	Activity (nmol/mg*min) ± SD
Bs3	DMSO	63.7 ± 2.6
Bs3	MMI	13.3 ± 2.2
Bs3	YUCASIN	3.4 ± 1.2
Bs3_S211A_	DMSO	137.2 ± 3.6
Bs3_S211A_	MMI	115.5 ± 0.9
Bs3_S211A_	YUCASIN	83.3 ± 3.1

### Competitive inhibitors of YUCs inhibit Bs3_S211A_ to a lesser extent than the Bs3 wildtype protein

We tested by *in vitro* assays if the two chemicals yucasin and methimazole (MMI), which are known competitive inhibitors of YUC function [25], had an influence on NADPH oxidation by recombinant Bs3 or Bs3_S211A_ proteins. We observed that both Bs3 and Bs3_S211A_ have reduced NADPH oxidase activity upon inhibitor treatment (Fig 4). Yucasin and MMI reduced the NADPH oxidase of Bs3 activity by 95% and 79%, respectively (Fig 4). These findings are in agreement with inhibitor studies on YUCs where yucasin was found to be a stronger inhibitor than MMI [25]. Competitive inhibitors bind to the active site of the enzyme and prevent the substrate from binding. Thus, our observation that competitive inhibitors of YUCs also inhibit Bs3 suggests that the substrate binding sites of YUCs and Bs3 are structurally similar. It is worth noting that yucasin and MMI reduced the NADPH oxidase activity of the Bs3-derivative Bs3_S211A_ by only 39% and 16%, respectively (Fig 4). The observation that both competitive inhibitors had less pronounced effects on Bs3_S211A_ as compared to the Bs3 wild-type protein suggests that the S211A mutation affects the topology of the substrate binding site of Bs3.

### Fusion of redox sensitive roGFP2 to Bs3 has no impact on function

**Fig 5:**
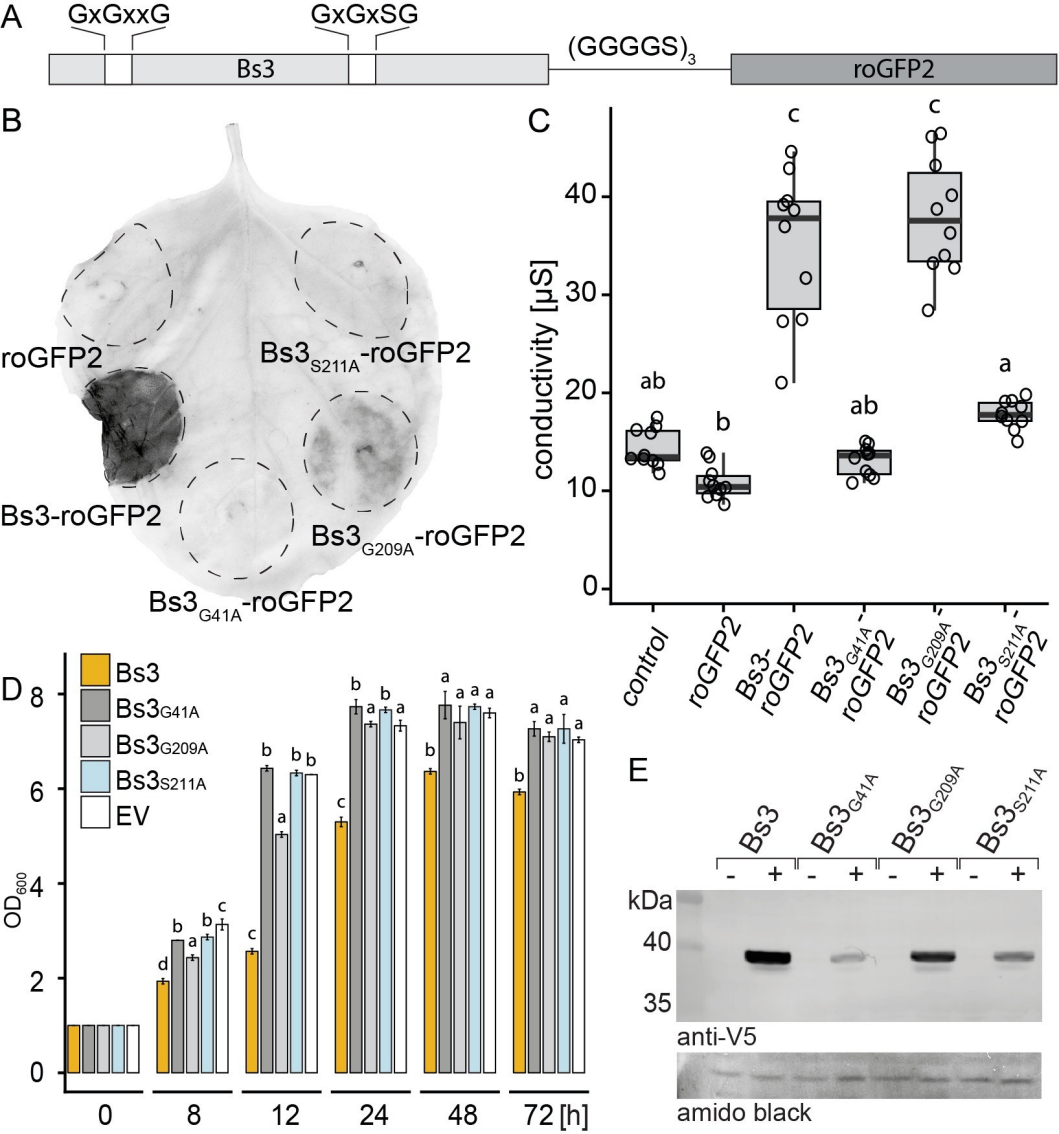
*35S* driven expression of *Bs3*_*G209A*_ triggers HR. A) Schematic representation of a Bs3-roGFP2 fusion constructs. Amino acid sequences above the dark-grey boxes indicate conserved sequences of FAD (left) and NADPH (right) binding sites. B) *Agrobacterium* strains carrying the indicated constructs under control of the *35S* promoter were infiltrated into *N*. *benthamiana*. Four days post infiltration, leaves were harvested and cleared with ethanol. HR is visible as dark spots. Dashed lines mark the infiltrated area. C) Ion leakage measurements of plant tissue expressing *roGFP2* and *Bs3-roGFP2* derivatives. Indicated constructs were expressed under control of the *35S* promoter in *N*. *benthamiana* leaves via *Agrobacterium*-mediated transient transformation. Three days post infiltration, leaf discs were cut and incubated in ultrapure water. Conductivity was measured after 20 hours of incubation. Boxplots represent values of 10 replicates. Single values are depicted as black circles. Different letters denote statistically significant differences (P < 0.05, ANOVA with posthoc Tukey Honest Significant Difference test) D) *Bs3* and *Bs3* mutant derivatives were cloned downstream of a galactose inducible promoter (*pGAL1*) and transformed into yeast. Yeast strains were grown in repressing medium overnight, diluted to OD_600_ = 1 in inducing medium and incubated at 28°C with shaking. Samples were taken at indicated timepoints and OD_600_ was measured. Values represent mean +/- SD of three replicates. E) Yeast cultures carrying the indicated constructs were grown in repressing (-) or inducing (+) liquid medium for six hours. Protein expression was monitored via an anti-V5 immunoblot. Amido black staining was used to visualize total protein load. Different letters denote statistically significant differences calculated for each timepoint (P < 0.05, ANOVA with posthoc Tukey Honest Significant Difference test).

The plant intracellular environment is likely distinct from the *in vitro* conditions in which we demonstrated NADPH oxidase activity of Bs3 resulting in H_2_O_2_ production (Fig 4). In particular, the putative metabolic substrate of Bs3 can be assumed to be present *in vivo*. Unfortunately, direct measurement of H_2_O_2_ and the differentiation from other ROS is not possible *in planta*. Therefore, we utilized the reduction-oxidation-sensitive GFP-derivative roGFP2 to measure increases in oxidation level, which are indicative of H_2_O_2_ production. Two cysteine residues in roGFP2 mediate its redox-sensitivity, which correlates with changes in its fluorescent properties ultimately allowing for ratiometric measurements in plant cells [26, 27]. To measure Bs3-dependent redox changes we translationally fused roGFP2 to Bs3, thereby positioning the redox-reporter in spatial proximity of Bs3 (Fig 5). We first tested if the redox reporter roGFP2 had an impact on HR induction when translationally fused to Bs3. *Bs3-roGFP* was expressed via agroinfiltration of *35S* promoter-driven T-DNA constructs in *N*. *benthamiana* leaves (Figs 5 and S2). Three days post inoculation (dpi), leaf discs were harvested for ion leakage assays which allow to monitor HR via an increase of conductivity. At four dpi, full leaves were harvested and cleared with ethanol to visualize the HR (Fig 5). *35S*-promoter-driven *Bs3-roGFP2* but not *roGFP2* induced visible cell death and high conductivity in *N*. *benthamiana* leaves, demonstrating that the translationally-fused roGFP2 reporter does not interfere with the Bs3-dependent HR (Fig 5). Overall, the observed *in planta* reactions induced by Bs3-roGFP2 fusion proteins are consistent with the previously observed phenotypes of corresponding Bs3-GFP fusion proteins (Fig 2, [13]).

### Phenotypes induced by Bs3 mutant derivatives are consistent in plants and yeast

To analyze how distinct mutations in cofactor binding sites affect activity of Bs3 *in planta*, the redox-reporter was fused to the Bs3-derivative Bs3_S211A_ and the previously studied Bs3 derivatives Bs3_G209A_ (mutation in NADPH binding site [Gx**G**xSG → Gx**A**xSG]) and Bs3_G41A_ (mutation in FAD binding site [Gx**G**xxG → Gx**A**xxG]; [13]). The *Bs3* mutant derivatives were expressed via agroinfiltration of *35S* promoter-driven T-DNA constructs into *N*. *benthamiana* leaves (Figs 5 and S2). At 3 dpi, *Bs3*_*G209A*_*-roGFP2* caused a similar increase in electrical conductivity as *Bs3-roGFP2*. At four dpi *Bs3*_*G209A*_*-roGFP2* induced a less distinct HR phenotype compared *Bs3-roGFP2*. The Bs3 mutant derivatives *Bs3*_*G41A*_*-roGFP2* and *Bs3*_*S211A*_*-roGFP2* did not induce HR or an increase in conductivity in agroinfiltration assays (Fig 5). To analyze if the graduated HR intensities, induced by Bs3 mutant derivatives *in planta* are reflected in yeast, we studied the effect of *Bs3*, *Bs3*_*G41A*,_ *Bs3*_*S209A*_ and *Bs3*_*S211A*_ expression on yeast grown in liquid medium. To do so, yeast cultures were diluted to a starting OD_600_ of 1 in inducing medium containing galactose and yeast growth was monitored in a time-course experiment over a period of three days (Fig 5). Strains containing the mutant derivatives Bs3_G41A_ and Bs3_S211A_, that both do not trigger HR *in planta*, showed no yeast growth inhibition. Strains containing Bs3 and Bs3_G209A_ that trigger HR *in planta*, show impaired growth. In accordance with the *in planta* phenotype, expression of *Bs3*_*G209A*_ causes less severe inhibition of growth compared to *Bs3* (Fig 5). Immunoblot analysis of *Bs3* and its derivatives in yeast revealed that Bs3 and Bs3_G209A_ proteins were similarly abundant, while the mutant derivatives Bs3_G41A_ and Bs3_S211A_ showed somewhat lower levels (Fig 5) [13]. Altogether, severity of *in planta* HR and inhibition of growth in yeast correlated consistently across all tested Bs3 derivatives.

### roGFP2 reporter assays indicate *in planta* oxidase activity for Bs3 and Bs3_S211A_

**Fig 6:**
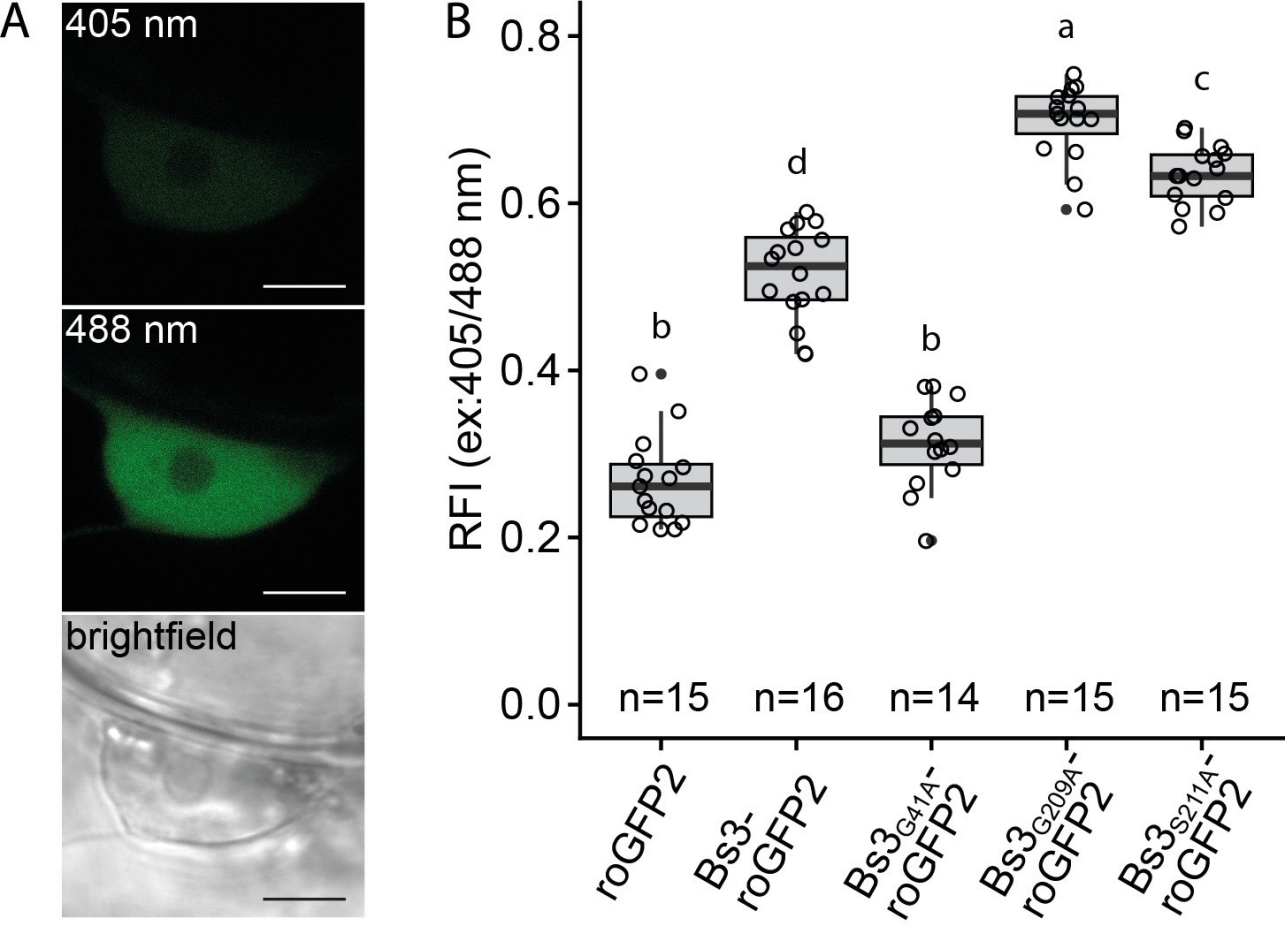
*In planta* oxidase activity of Bs3 and its derivatives. A) Representative pictures of roGFP2 fluorescence in the nucleus with excitation at 405 and 488 nm. Scale bar = 5 μm B) RoGFP2 oxidation is increased in *Bs3*, *Bs3*_*G209A*_ and *Bs3*_*S211A*_ expressing leaf tissue. Indicated constructs were expressed under control of the *35S* promoter in *N*. *benthamiana* via *Agrobacterium*-mediated transient transformation. 30 hpi leaf discs were analysed by ratiometric laser scanning microscopy and the ratios of fluorescence intensity (RFI) upon excitation at 405 and 488 nm was determined. n = number of observations. Single values are depicted as black circles. Different letters denote statistically significant differences (P < 0.05, ANOVA with posthoc Tukey Honest Significant Difference test).

To analyze changes of the intracellular oxidation state caused by Bs3 and its mutant derivatives, the different *roGFP2* fusion constructs with full, reduced and no capacity to trigger HR (*Bs3-roGFP2/ Bs3*_*G209A*_*-roGFP2/ Bs3*_*G41A*_*-roGFP2 and Bs3*_*S211A*_*-roGFP2*) and *roGFP2* were expressed in *N*. *benthamiana* leaves (Figs 6 and S2). Thirty hours post inoculation (hpi) confocal laser scanning microscopy (CLSM) was used to determine the fluorescence intensity upon excitation at 405 and 488 nm (Fig 6). Oxidation of roGFP2 causes an increase of fluorescence at 405 nm excitation and a corresponding decrease at 488 nm excitation. Therefore, a higher relative fluorescence intensity (ratio 405nm/ 488nm) is indicative for a higher oxidation state. We found that the HR-inducing proteins Bs3-roGFP2 and Bs3_G209A_-roGFP2 had about two-fold higher RFI values than the roGFP control (Fig 6). This observation suggests that Bs3 and Bs3_G209A_ have indeed NADPH oxidase activity *in planta*, which would be consistent with a model where ROS produced by Bs3 triggers HR. Bs3_G41A_-roGFP2, which does not trigger HR, had similar RFI values as the roGFP2 control, is still in agreement with our proposed model. However, the NADPH-binding site mutant Bs3_S211A_-roGFP2, which does not trigger HR *in planta*, had higher RFI values than the HR inducing Bs3-roGFP2 fusion protein. Indeed, the elevated oxidation state of roGFP2 induced by Bs3_S211A_ *in planta* is consistent with our *in vitro* studies that showed increased NADPH oxidase activity in Bs3_S211A_ as compared to the wildtype Bs3 protein (Figs 4 and 6). In summary, we found that the mutant derivative Bs3_S211A_ does not trigger HR, but induced a similar increase of the intracellular oxidation compared to the Bs3 wild-type protein, which suggests that the putative release of H_2_O_2_ by Bs3 is not sufficient to trigger the Bs3-dependent HR.

## Discussion

### Competitive inhibitors of YUCCA proteins inhibit Bs3

Pepper Bs3 is one out of six currently known executor R proteins [2, 4–8, 28]. Bs3 is exceptional since it is the only executor that shares sequence homology to proteins of known function. Due to the sequence homology of Bs3 to YUCCA proteins, which catalyze conversion of IPA to IAA, it would seem plausible that the enzymatic capabilities of Bs3 and YUCCAs are similar. The 70 amino acid long stretch that is present across all YUCCAs but absent from Bs3 (S1 Fig) might contain domains that mediate metabolic feedback regulation of YUCCAs. For example, one could envision that in absence of IPA the production of H_2_O_2_ by YUCCAs would stop enzymatic activity YUCCAs before H_2_O_2_ accumulates to cytotoxic levels. Accordingly, the lack of this 70aa stretch in Bs3 will possibly cause accumulation of Bs3-dependent metabolites to cytotoxic levels.

Previously, we showed that Bs3, in contrast to the related YUCCA proteins, does not synthesize IAA and ultimately that Bs3-dependent HR does not involve changes in IAA levels [13]. While Bs3 and YUCCAs seem to differ in their metabolic product, it would seem possible that both enzymes use IPA as a metabolic substrate. Unfortunately, IPA is a rather unstable metabolite that spontaneously converts into IAA *in vitro* [29]. In consequence, quantification of IPA consumption by recombinant Bs3 protein *in vitro* proves to be rather challenging. However, the previously identified competitive inhibitors of YUCCA proteins that reduce enzymatic activity by binding to the substrate binding pocket provide an alternative means to compare metabolite binding sites of Bs3 and YUCCA proteins. Yucasin and methimazole, which are both competitive inhibitors of YUCCA proteins [25], reduced NADPH oxidase activity of both Bs3 and Bs3_S211A_ (Fig 4) thereby suggesting that Bs3 contains a substrate binding site that is structurally related to that of YUCCAs. The less severe inhibition of Bs3_S211A_ compared to Bs3 by yucasin and methimazole is in line with the notion that the S to A mutation does change the substrate-binding site but still allows NADPH oxidation. Reduced affinity of Bs3_S211A_ to a metabolic substrate would favour C4a break down without substrate oxygenation and would explain the increased NADPH oxidase activity of this particular Bs3 mutant derivative.

### H_2_O_2_ production by Bs3 is not sufficient to trigger HR

Based on the fact that YUCCAs and other FMOs can release substantial amounts of H_2_O_2_ by oxidizing NADPH without substrate conversion in a process referred to as “uncoupling” [16, 30], we hypothesized that Bs3 would not bind a substrate but induce HR by H_2_O_2_ production. We envisioned two possible ways in which Bs3 could trigger HR by the production of H_2_O_2_. Firstly, Bs3 might produce excessive amounts of H_2_O_2_ that cause oxidative damage to the plant cell, or alternatively, Bs3 produces H_2_O_2_ as a signaling molecule that activates a cascade leading to plant defense and HR. Indeed, our *in vitro* studies revealed that recombinant Bs3 protein produces substantial amounts of H_2_O_2_ (Fig 4), and our analysis of the intracellular oxidation state by roGFP2-based reporter assays (Fig 6) suggests that Bs3 releases H_2_O_2_ not only *in vitro* but also *in vivo*.

To reinforce the possible causal link between direct production of H_2_O_2_ by Bs3 and HR we studied the function of Bs3 mutant derivatives. For example, the Bs3 mutant derivative Bs3_G41A_, which based on roGFP2 reporter assays, does not produce H_2_O_2_ (Fig 6) did also not trigger HR *in planta* (Fig 5), being consistent with a model in which H_2_O_2_ produced by Bs3 triggers HR. To further support this hypothesis, we replicated a mutation in the fungal FMO SidA that was previously shown to cause increased NADPH oxidase activity [20]. Indeed, *in vitro* and *in vivo* studies of the Bs3_S211A_ mutant showed increased NADPH oxidase activity and H_2_O_2_ production (Figs 4 and 6). Yet, while Bs3_S211A_ has higher oxidase activity than the wild-type Bs3 protein, it does not trigger HR *in planta* (Figs 1 and 5). This implies that release of H_2_O_2_ by Bs3 is not sufficient to trigger HR. We therefore postulate, that Bs3 converts a metabolite which is possibly structurally related to IPA into a yet to be identified product that triggers HR. Whether or not this metabolic product of Bs3 is simply cytotoxic or if the metabolic product acts as a defense signaling molecule remains to be seen.

### Differentiation of direct and indirect ROS production during Bs3 HR

We studied H_2_O_2_ synthesis during Bs3 HR *in planta*. The results of our measurements via the roGFP2 redox reporter and by DAB staining strongly suggest that the roGFP2 reporter and DAB staining detect distinct H_2_O_2_ pools. While roGFP2 based measurements revealed high intracellular oxidation states for both, Bs3 and Bs3_S211A_ (Fig 6), only expression of Bs3 but not Bs3_S211A_ produced brown precipitates in DAB staining being indicative for accumulation of ROS (Fig 2). The direct correlation of HR with DAB staining but not the roGFP2 reporter readout indicates that the H_2_O_2_ detected via DAB staining was not produced directly by Bs3, but originated from other sources like membrane-bound NADPH oxidases or apoplastic peroxidases that are activated in the course of Bs3 induced HR. This is supported by the finding that the intensity of DAB staining is independent of Bs3 protein amount [13]. The DAB staining intensity increased until the onset of cell death, even though a decrease of Bs3 protein levels can be observed in *N*. *benthamiana* at 48 hpi (Fig 2, [13]).

In contrast to the DAB staining, ratiometric measurements are independent of expression levels and our roGFP2 based measurements were able to detect changes in the oxidation state as early as at the onset of Bs3-roGFP2 protein accumulation. RoGFP2 studies were carried out at 30 hpi, a timepoint at which no DAB staining was visible (Fig 2). The higher oxidation values measured for Bs3-roGFP2 compared to roGFP2 suggests that low amounts of H_2_O_2_ are produced directly by Bs3. The higher oxidation values obtained for Bs3_S211A_-roGFP2 show that these low amounts of H_2_O2 –presumably produced via the uncoupled reaction–are not sufficient to trigger cell death. However, while H_2_O_2_ produced by a Bs3 uncoupling reaction is not sufficient to trigger cell death it cannot be excluded that local changes in the oxidation state contribute to a signaling cascade that results in execution of HR.

### Bs3 derivatives that trigger HR *in planta* consistently inhibit proliferation in yeast cells

We found that expression of *Bs3* limits proliferation of yeast cells, which are particularly amenable to genetic screens and can be used to dissect the Bs3-triggered cell death reaction in the future. The functional analysis of Bs3 and derivatives thereof in the plant- and yeast systems were consistent in all cases. For example, Bs3 and the NADPH-mutant derivative Bs3_G209A_ triggered HR *in planta* (Fig 5) and also inhibited proliferation of yeast cells (Fig 5). Bs3_G209A_ induced a somewhat weaker HR than the wildtype Bs3 protein *in planta* (Fig 5) and the same trend was observed in the yeast growth assay where expression of Bs3_G209A_ or Bs3 induced moderate and strong inhibition of yeast growth, respectively. Moreover, the Bs3 derivatives Bs3_G41A_ and Bs3_S211A_, did not trigger HR *in planta* and also failed to inhibit proliferation of yeast cells (Fig 5). Comparison of expression levels of Bs3 and derivatives in yeast seem to indicate that cell-death inducing Bs3 derivatives show generally higher expression levels as the Bs3 derivatives that do not trigger growth arrest (Fig 5). However, *in planta* expression levels of the cell inducing Bs3 wt protein were equal or lower than expression levels of Bs3_S211A_ that does not trigger HR *in planta*. In summary these observations demonstrate that capability of the studied Bs3 derivatives to induce cell death can not be explained by variation in protein expression levels but resembles the biochemical properties of the studied protein variants. The observed consistency of the Bs3-dependent phenotypes in yeast and plant cells possibly suggest that both phenotypes have a common molecular basis. Therefore, it stands to reason that the observed phenotypes in yeast and plants are caused by the same metabolite that Bs3 presumably produces in plant and yeast cells.

### Recombinant Bs3 protein as a tool to uncover the metabolic basis of the Bs3-triggered HR

Previously conducted biochemical studies uncovered that recombinant Arabidopsis YUCCA6 protein uses NADPH and oxygen to convert IPA to IAA [10]. Here, we established a protocol for purification of enzymatically active Bs3 protein that now enables us to compare enzymatic activity of Bs3 and the YUCCA proteins by *in vitro* assays. Given their high structural relatedness, it seems likely that Bs3 and YUCCA proteins are also similar with respect to their enzymatic features. Indeed, our studies revealed that Bs3, just like YUCCA6, has NADPH oxidase activity (Fig 4). Moreover, inhibitor studies suggest that YUCCA proteins and Bs3 have the same or at least structurally related metabolic substrates (Fig 4). We envision future studies where incubation of metabolic candidate substrates, with enzymatically active Bs3 protein could provide the possibility to identify metabolic products of Bs3 by mass spectrometry. In summary, the established protocol for purification of recombinant enzymatically active Bs3 protein is the first step towards exploitation of its biochemical properties and holds the key to uncover the basis of Bs3-triggered immune reactions by metabolic studies.

## Material and methods

### Plants and growth conditions

*N*. *benthamiana* and *Capsicum annuum* (cultivar ECW123) plants were grown at 20–24°C at 35–60% humidity with a light intensity of 12,3 klx and 16 hours light/ 8 hours dark cycle. Four to six weeks old plants were used for experiments.

### Plasmid construction

For *in planta* expression, the *Bs3* CDS was assembled with a *35S* promoter and *eGFP* into the *LIIa* expression vector via Golden Gate cloning [31]. The serine to alanine mutation was introduced by PCR (PrimerFW: P-GCCGGGATCGATATCTCACTTG PrimerREV: ATTGCCACAGCCAACCGC). For bacterial expression, the Bs3 coding sequence was cloned into the pET-53-DEST (Novagen) expression vector via Gateway cloning. Protein solubility could be dramatically improved by deletion of the nucleotides encoding for the recombination site and the codons accounting for the two N-terminal methionines of Bs3. Deletion of these nucleotides was done by PCR mutagenesis (PrimerFW: P-GTGATGGTGGTGGTGATGTG and PrimerREV: AATCAGAATTGCTTTAATTCTTGTT CAC). For expression in yeast, the Bs3 CDS was cloned into the pYES-DEST-52 vector via Gateway cloning without further modification.

### DAB staining

Leaves are vacuum infiltrated with DAB staining solution (10 mM Sodium phosphate, 1 mg/ml Diaminobenzidine-tetrahydrochloride, 0.1% Tween-20, pH = 7.2), incubated at room temperature with gentle shaking for at least 5 hours and subsequently de-stained with 80% ethanol at 60°C.

### *Xanthomonas* and *Agrobacterium* infiltration

*Xanthomonas* (82–8 uns*) carrying the pDSK602 vector with either *AvrBs3* or *AvrBs2* were grown at 28°C in NYG medium (5 g/L peptone, 3 g/L yeast extract, 20 g/L glycerol) containing Rifampicin and Spectinomycin at a final concentration of 100 μg/ml for 1 day. Cultures were pelleted and re-suspended in water to OD_600_ = 0.4. Leaves of *C*. *annuum* were infiltrated with a blunt end syringe from the abaxial side.

*Agrobacterium tumefaciens* (GV3101) carrying the respective binary plasmids were grown over night at 28°C in YEB medium (5 g/L beef extract, 1 g/L yeast extract, 5 g/L peptone, 5 g/L sucrose, and 0.5 g/L mM MgSO_4_, pH 7.2) containing Rifampicin and Spectinomycin at a final concentration of 100 μg/ml. Cultures were pelleted and resuspended in water to OD_600_ = 0.4. Leaves of *N*. *benthamiana* were infiltrated with a blunt end syringe from the abaxial side.

### Protein expression

*E*. *coli* Rosetta (Novagen) were transformed with *pET-53-DEST_Bs3* or derivatives, plated on LB Agar containing Ampicillin (100 μg/ml) and Chloramphenicol (15 μg/ml) and incubated at 37°C over night. Several colonies were pooled to inoculate LB medium (10 g/L NaCl, 5 g/L yeast extract, 10 g/L tryptone) supplemented with Ampicillin (100 μg/ml) and Chloramphenicol (15 μg/ml). After over night incubation at 37°C and 180 rpm, this starter culture was used to inoculate 2 L TB Medium (24 g/L yeast extract, 20 g/L tryptone, 4 ml/L glycerol, 0.072 M K_2_HPO_4_, 0.017 M KH_2_PO_4_) supplemented with Ampicillin (100 μg/ml) at a starting OD_600_ of 0.05. The culture was incubated at 37°C with shaking at 120 rpm until it reached an OD_600_ of 1. Cultures were then cooled down for 15 min in ice water and protein expression was induced with a final concentration of 1 mM Isopropyl 1-thio-β-D-galactopyranoside (IPTG). The cells were incubated for another 2,5 h at 18°C with shaking and followed by centrifugation (4500 x g, 30 min, 4°C). Pellets were stored at -20°C until further use.

### Protein purification

The bacterial pellet was re-suspended in lysis buffer (50 mM Potassium phosphate, 10% glycerol, 30 mM Imidazole, 1% Tween, protease inhibitor, 1 μM FAD, 1 mM DTT, pH = 8) using 5 ml of buffer per gram of pellet. 30 ml cell suspension were sonicated for 5 min (5s on/10s off, 60% amplitude) with a sonicator (EpiShear, Active Motif) equipped with a ¼” microtip probe. The lysate was centrifuged (16000 x g, 4°C) for 30 min to pellet cell debris. An ÄKTA Pure 25 FPLC system, equipped with a 5 ml HisTrapFF Crude Column (GE Healthcare), was used for affinity purification. After column equilibration with 10 column volumes (CV) of wash buffer (50 mM Potassium phosphate, 10% Glycerol, 30 mM Imidazole, pH = 8), the supernatant was loaded onto the column and washed with 20 CV wash buffer. The protein was eluted with 2 CV elution buffer (50 mM Potassium phosphate, 10% Glycerol, 500 mM Imidazole, pH = 8). Imidazole was removed by dialysis and the protein was frozen in liquid nitrogen and stored at - 80°C until further use.

### NADPH oxidation

Spectroscopic assays were carried out in quartz cuvettes with a UV-900 UV-vis spectrometer (Shimadzu) equipped with a temperature controlled cell holder (TCC-100) set to 25°C. Samples were diluted with 50 mM potassium phosphate buffer (pH = 8) containing NADPH. Exact NADPH concentrations were calculated from its absorption and the extinction coefficient at 340 nm (ε_340_ = 6220 M^-1^ cm^-1^).

### Detection of H_2_O_2_ with HyPerBlu

Protein was mixed with NADPH solution (100 μM) and incubated at RT for 15 min. Per replicate, 5 μl of this solution were transferred to a white 385 well plate, mixed with 5 μl of HyPerBlu solution (Lumigen) and incubated in darkness for 15 min at RT. Subsequently, luminescence was measured using a Berthold Tristar LB 941 plate reader. H_2_O_2_ concentrations were calculated using a standard curve prepared with known concentrations of H_2_O_2_.

### Redox reporter microscopy

Constructs in which *roGFP2* [26] was fused to *Bs3* and its derivatives were transiently expressed in four to six week old *N*. *benthamiana* plants via *Agrobacterium* mediated transient transformation. 30 hpi, images were acquired using a Leica TCS SP8 confocal microscope by successive excitation at 405 nm and 488 nm and emission at 498 to 548nm. Images of nuclei were taken using a 63x water immersed objective and 10x digital magnification. Argon laser intensity was adjusted in a way so that pixels were close to saturation in samples with highest *roGFP2* expression. UV laser intensity was adjusted allowing imaging of samples with lowest *roGFP2* expression. Fiji [32] was used to crop the surrounding of the nuclei and to calculate mean pixel intensity of the fluorescent area.

### Ion leakage measurements

Ion leakage measurements were conducted using the CM100-2 conductivity meter (Reid & Associates). Each well was filled with 1 ml ultrapure water. Leaf discs (Ø 4 mm) were harvested three days post infiltration. One disc was added per well and incubated at RT. Ion leakage was measured after 20 hours of incubation.

## Supporting information

S1 Fig Alignment of *Capsicum annuum* Bs3 (CaBs3), *Capsicum annuum* YUCCA (CaYUC) and *Arabidopsis thaliana* YUCCA (AtYUC) proteins. The ~70 amino acid sequence that is absent from Bs3 in comparison to YUCs is highlighted in blue. The conserved FAD and NADPH binding sites (GxGxxG) are indicated. The red box highlights the conserved serine within the NADPH binding site.
(TIF)

S2 Fig Localization of Bs3 and Bs3 derivatives fused to roGFP2. Indicated constructs were expressed in *N*. *benthamiana* leaves via *Agrobacterium*-mediated transient transformation. Leaf discs for microscopy were cut at 30 hpi. Pictures show GFP fluorescence (upper row) and brightfield (lower row).
(TIF)

S1 File (PDF)
