# Supplementary figures and images for: The flavin monooxygenase Bs3 triggers cell death in plants, impairs growth in yeast and produces H2O2 in vitro
Source: PLoS One. 2021 Aug 19;16(8):e0256217. doi: 10.1371/journal.pone.0256217 (PMC8375990; doi:10.1371/journal.pone.0256217)

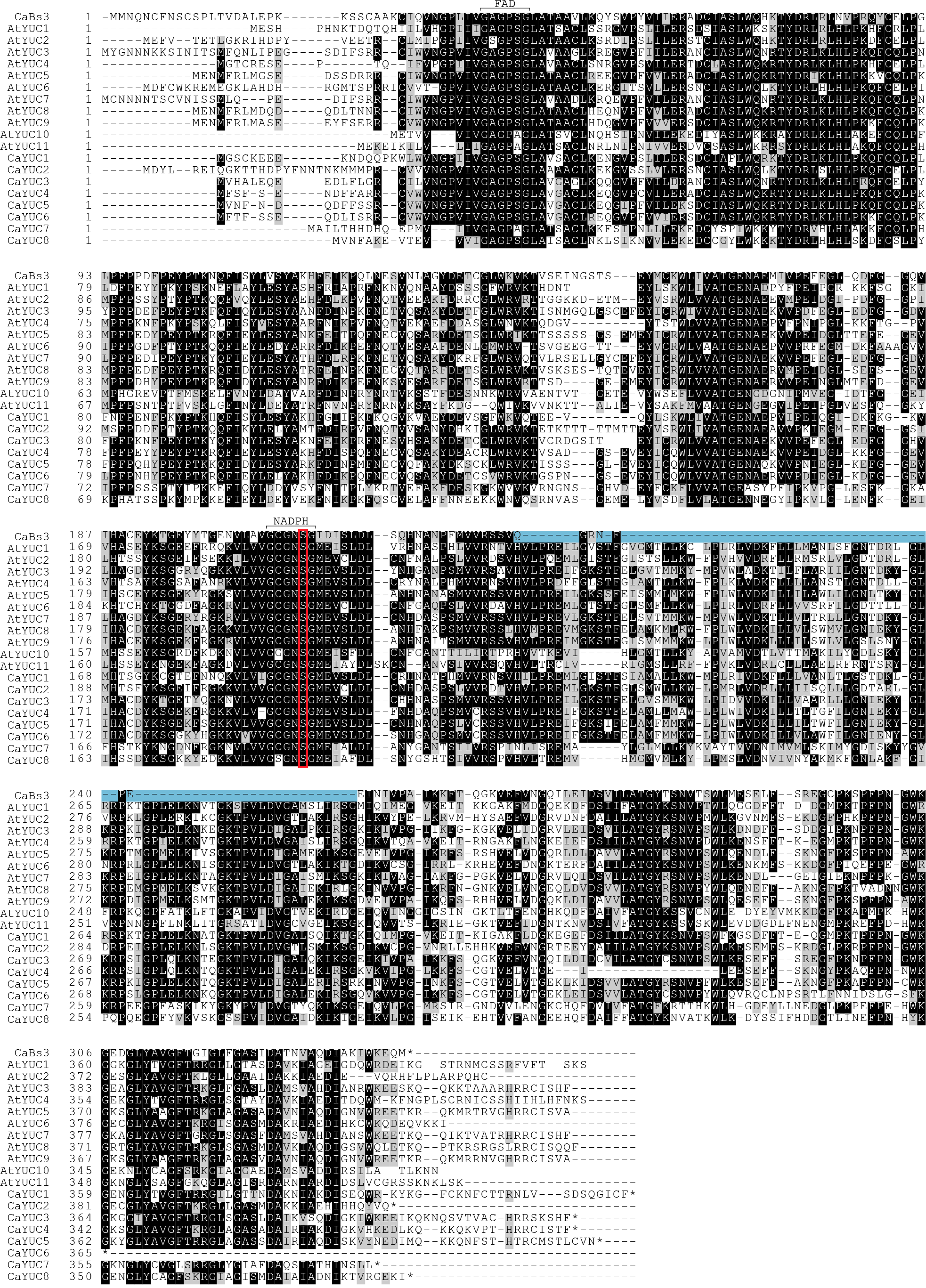

Supplement: S1 Fig — The ~70 amino acid sequence that is absent from Bs3 in comparison to YUCs is highlighted in blue. The conserved FAD and NADPH binding sites (GxGxxG) are indicated. The red box highlights the conserved serine within the NADPH binding site. (TIF) [file pone.0256217.s001.tif]

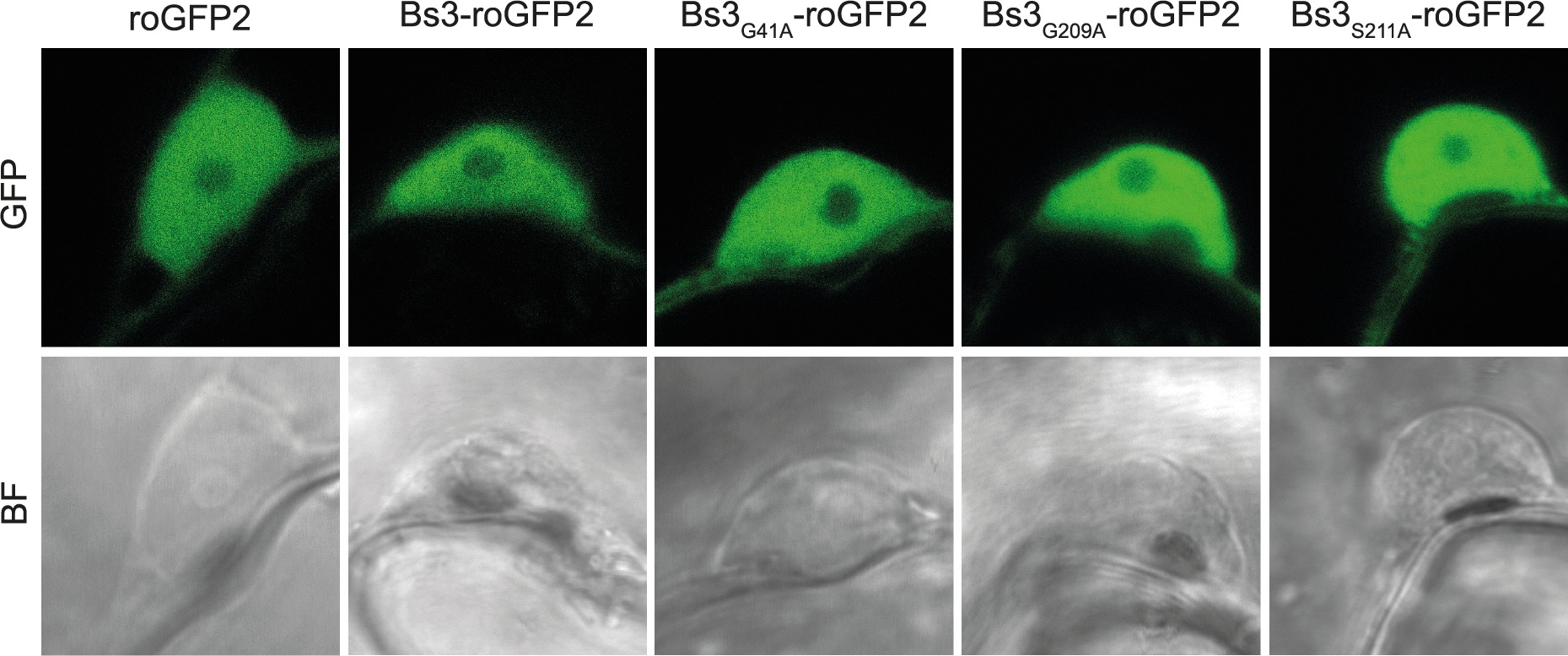

Supplement: S2 Fig — Indicated constructs were expressed in N. benthamiana leaves via Agrobacterium-mediated transient transformation. Leaf discs for microscopy were cut at 30 hpi. Pictures show GFP fluorescence (upper row) and brightfield (lower row). (TIF) [file pone.0256217.s002.tif]
