# Supplementary material for: The flavin monooxygenase Bs3 triggers cell death in plants, impairs growth in yeast and produces H2O2 in vitro
Source: PLoS One. 2021 Aug 19;16(8):e0256217. doi: 10.1371/journal.pone.0256217 (PMC8375990; doi:10.1371/journal.pone.0256217)
Supplement: S1 File — (PDF) [file pone.0256217.s003.pdf]

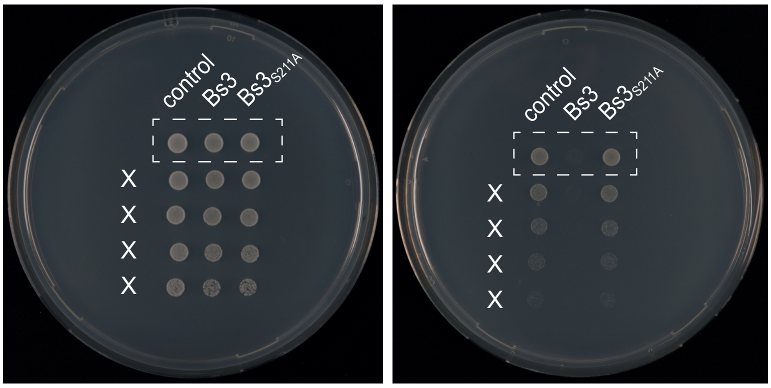

Identity of experimental samples: Yeast culture dropped on solid medium
Method used to capture the image: Plates were photographed with a Canon EOS 750 D camera
Figure panel generated from this original image: Figure 1A

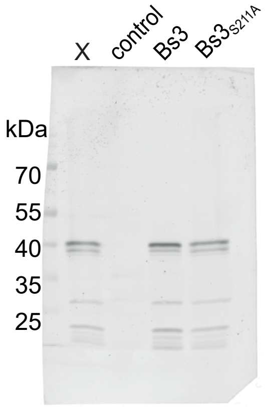

Identity of experimental samples: Western blot from PAGE separated yeast culture samples
Method used to capture the image: Fluorescent signal of secondary antibodies was recorded with an Amersham Typhoon scanner equippedwith a BPFR 700 filter at 680 nm
Figure panel generated from this original image: Figure 1A

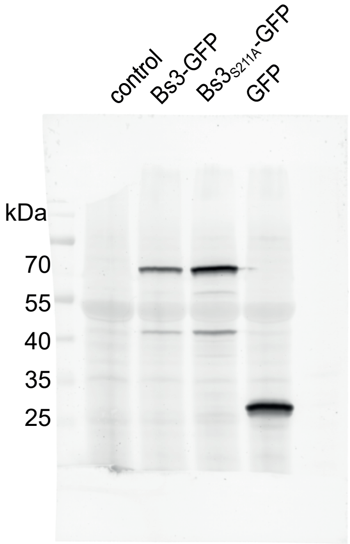

Identity of experimental samples: Western blot from PAGE separated plant leaf samples
Method used to capture the image: Fluorescent signal of secondary antibodies was recorded with an Amersham Typhoon scanner equippedwith a BPFR 700 filter at 680 nm
Figure panel generated from this original image: Figure 1C

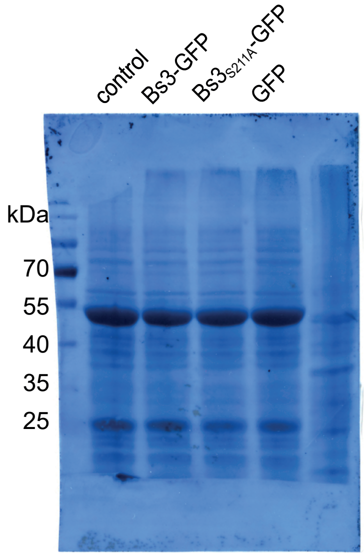

Identity of experimental samples: Western blot from PAGE separated plant leaf samples
Method used to capture the image: Amido black stained membrane was scanned with an EPSON V700 photo flatbed scanner.
Figure panel generated from this original image: Figure 1C

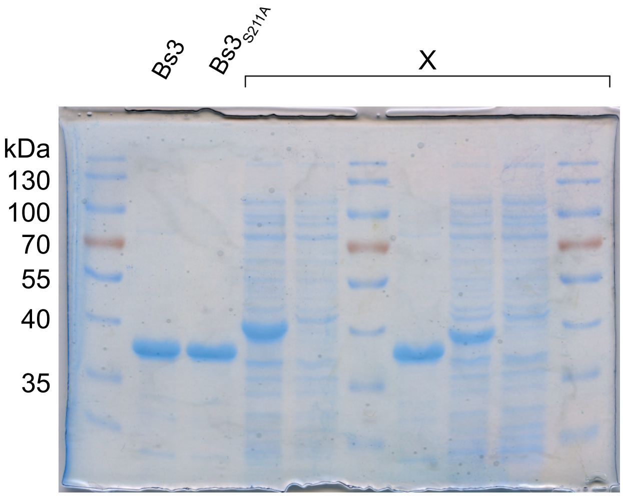

Identity of experimental samples: PAGE of purified protein
Method used to capture the image: The gel was scanned with an EPSON V700 photo flatbed scanner.
Figure panel generated from this original image: Figure 3

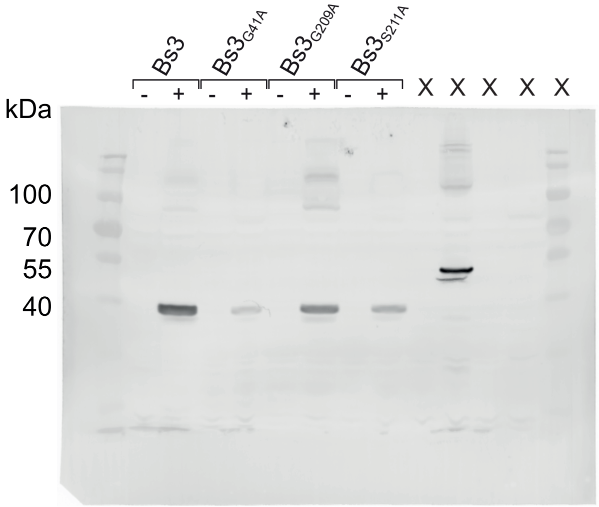

Identity of experimental samples: Western blot from PAGE separated yeast culture samples
Method used to capture the image: Fluorescent signal of secondary antibodies was recorded with an Amersham Typhoon scanner equippedwith a BPFR 700 filter at 680 nm.
Figure panel generated from this original image: Figure 5E

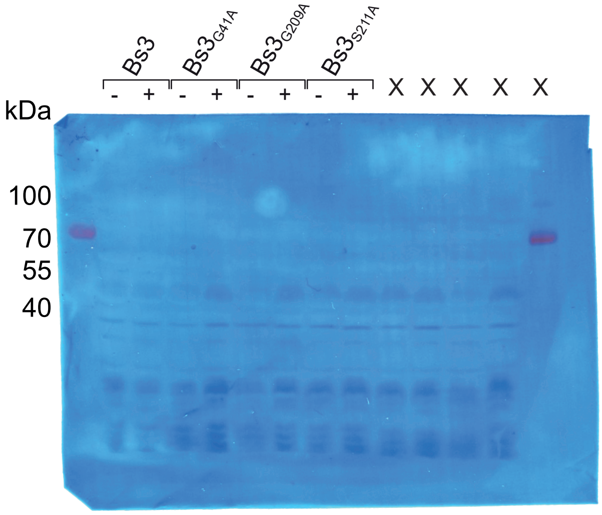

Identity of experimental samples: Western blot from PAGE separated yeast culture samples
Method used to capture the image: Amido black stained membrane was scanned with an EPSON V700 photo flatbed scanner.
Figure panel generated from this original image: Figure 5E
